# Effect of Heat Shock Preconditioning on Pressure Injury Prevention via Hsp27 Upregulation in Rat Models

**DOI:** 10.3390/ijms23168955

**Published:** 2022-08-11

**Authors:** Huiwen Xu, En Takashi, Jingyan Liang, Yajie Chen, Yuan Yuan, Jianglin Fan

**Affiliations:** 1 Division of Basic & Clinical Medicine, Faculty of Nursing, Nagano College of Nursing, Komagane, Nagano 399-4117, Japan; 2 School of Nursing & Public Health, Yangzhou University, Yangzhou 225000, China; 3 Institute of Translational Medicine, Medical College, Yangzhou University, Yangzhou 225000, China; 4 Department of Molecular Pathology, University of Yamanashi, 1110 Shimokato, Tokyo 409-3898, Japan

**Keywords:** pressure injury, hydrotherapy, preconditioning, prevention, Hsp27

## Abstract

Pressure injury (PI) prevention is a huge industry and involves various interventions. Temperature and moisture are important factors for wound healing; however, the active mechanism by which “moist heat” affects PI prevention has not yet been clarified. Thus, we explored the protective and therapeutic effects of hydrotherapy on PI based on the preconditioning (PC) principle, which might be useful for clinical practice. This study aimed to investigate the preventive mechanisms of heat shock preconditioning on PIs in rat models. The experiment was performed in the basic medical laboratory of Nagano College of Nursing in Japan. Ten rats were divided into two groups, with five rats in each group. Rats in the control group were not bathed. Rats in the preconditioning group (PC group) were bathed with hot tap-water. Bathing was conducted thrice a week. After bathing for 4 weeks, the PI model was constructed on the rats’ dorsal skin. The skin temperature, skin moisture, and area of ulcers were compared between the two groups. In vitro, we investigated the expression of heat shock protein 27 (Hsp27) in 6, 12, and 24 h after the PI model was constructed through Western blot analysis. Ulcers occurred in the control group 24 h after the PI model constructed, wheras the PC group exhibited ulcers after 36 h. The ulcer area was larger in the control group than that in the PC group after 24 h (all *p* < 0.05). The temperatures of PI wounds in the control group decreased and were lower than those in the PC group after 1, 6, 12, 36, and 48 h (all *p* < 0.05). However, the skin moisture levels of PI wounds increased in the control group and were higher than those in the PC group at the same time (all *p* < 0.05). Using Western blot analysis, hydrotherapy preconditioning showed the potential to increase Hsp27 expression after pressure was released (*p* < 0.05). We determine that heat shock preconditioning had a preventive effect on PIs in rat models, a result that may be associated with their actions in the upregulation of Hsp27.

## 1. Introduction

Pressure injury (PI) is a localized damage to the skin and/or underlying soft tissue, and it usually develops over bony prominence because of pressure in tandem with shear and/or friction [1]. Internationally, the prevalence rates of PI range from 2.2% to 42.7%, and incidence rates of PI vary between 1.4–49% [2]. PI treatment is difficult, and it may extend hospitalization and increase mortality and morbidity rates because of development of infectious complications [3]. Severe pain and discomfort reduce the quality of life of patients [4]. Treatment for PI is a great financial burden for both the family and society. The Healthcare Cost and Utilization Project estimated that the average cost of the treatment per patient for pressure injuries is USD 37,800 [5]. Additionally, the failure of a PI to heal or improve is associated with an increased death rate in nursing home residents [6,7,8]. In clinical practice, the PI incidence rate is an important nursing quality indicator [9,10]. Therefore, the effective prevention of PIs is important to reduce patient harm and medical costs, lessen morbidity and mortality, and improve nursing quality.

The concept of preconditioning was first introduced 30 years ago. It is a manifestation of hormesis. Hormesis is a biphasic dose response phenomenon in which exposure of a cell or organism to a low dose of a chemical agent or condition induces beneficial stimulation or adaptive effects, whereas higher doses cause inhibition or toxic effects [11,12]. This response to low doses of stress is considered an adaptive stress response following an initial disruption in homeostasis, enhancing an organism’s ability to withstand more severe stress [11,12]. Hormesis can potentially create biological shields to protect against more substantial and damaging challenges and minimize detrimentally and possibly life-threatening harm. [13] Preconditioning applies the concept of hormone biology by introducing one or more transient sublethal stimulus fragments to activate endogenous protective mechanisms against subsequent lethal injury [14]. Various preconditioning strategies have been tested to increase cell survival rates in damaged tissues.

Heat therapy is considered a potential hormetic intervention. Heat preconditioning is a powerful adaptive and protective phenomenon in which the organisms or cells previously exposed to sublethal acute heat stress display greater resistance to the effects of a subsequent, more potent stress [15]. Mild heat stress induces the heat shock response (HSR), which protects cells and organisms from severe damage [16]. HSR is characterized by the induction of molecular chaperones following a sudden increase in temperature [17], resulting in the synthesis and release of heat shock proteins (HSPs). Many HSPs prevent protein aggregation and participate in refolding or elimination of misfolded proteins in their capacity as chaperones [18]. Some HSPs, such as Hsp27, Hsp70 and Hsp90, can alleviate apoptosis and protect cells through preconditioning [19,20]. Heat preconditioning plays an important role in the prevention of PIs by improving hemodynamic abnormalities [21].

Hydrotherapy is an effective heat transmitter. In comparison with dry heat, moist heat has approximately 30% higher blood flow response than dry heat for warming the skin [22,23,24]. Burke and colleagues measured the efficacy of whirlpool therapy in pressure injury treatment and reported that the healing rate of the whirlpool group was considerably higher compared with the non-whirlpool [25].

As a traditional therapy, hydrotherapy is a non-invasive, safe, and efficient treatment. It mainly uses the temperature, mechanical, and chemical stimulation produced by warm water to treat and prevent disease in the human body. Clinical practice has shown that hydrotherapy’s obvious beneficial effects on the treatment of distal radius fractures [26], musculoskeletal injuries [27], and chronic and acute skin diseases [28]. Hydrotherapy is particularly useful for rheumatic pathologies since it promotes tissue anabolism and inhibits catabolism and inflammation [29]. In addition, hydrotherapy has been proven the safety and efficacy for damage prevention. In humans, a warm bath before exercise can prevent enthesopathy [30]. In rats, bathing for 15 min in hot plain water (40–42 °C) contributed to cardioprotection against ischemia injury [31] Furthermore, there were no adverse effects on organ health [32].

A previous study indicated that hydrotherapy could effectively prevent PI [33]. Shuwen et al. [33] explored the prevention effects of Baxianxiaoyao decoction bathing on rat PIs. They divided hairless rats into three groups, i.e., the control, the hot water bath, and the traditional Chinese medicine bath groups, and their results showed that bathing with hot tap water and traditional Chinese medicine were both effective in preventing PIs. However, the active mechanism of the effect on PI prevention has not been clarified. Our pre-experiment indicated that Hsp27 upregulated after hydrotherapy. The present study was conducted to confirm the protective effects of heat preconditioning against PIs using rat models and furthermore, to clarify the active mechanism of the PI-prevention effect by Western blot analysis.

## 2. Results

### 2.1. Macroscopic Observation of PI Wounds

**Figure 1:**
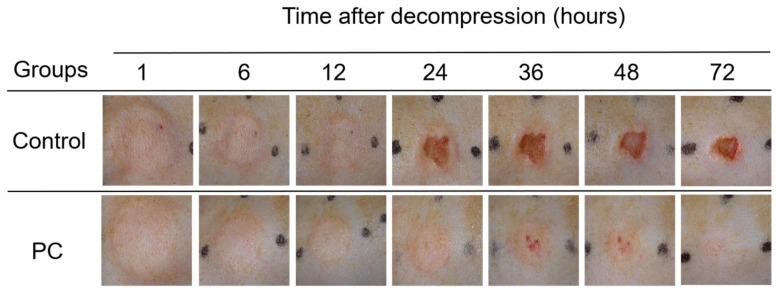
Photographs of PI wounds in two groups from 1 to 72 h after decompression. PI: pressure injury. PC: preconditioning.

**Figure 2:**
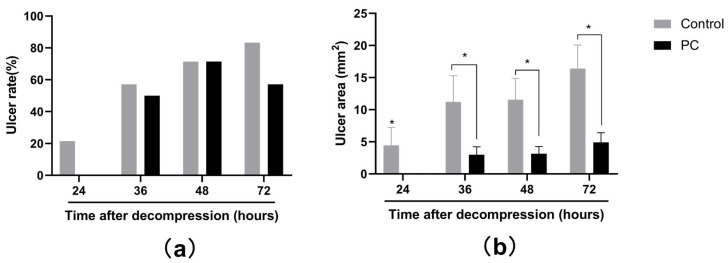
Ulcer rate (**a**) and ulcer area (**b**) after decompression in two groups. * *p* < 0.05. PC: preconditioning.

After pressure was released, the skin surface displayed edema and became gradually red; however, ulcers developed differently in the two groups. The skin was intact until 24 h and 36 h in the control and PC groups, respectively. Moreover, after decompression from 1 h to 72 h, the area of the ulcers was larger in the control group than that in the PC. The incidences of ulcers were presented by ulcer rate, which was calculated by the following formula: Ulcer rate = number of ulcers/number of PI model. Figure 1 and Figure 2a demonstrated that ulcers developed at 24 h and 36 h after decompression in the control and PC groups, respectively. In addition, a larger ulcer area was observed in the control group. The rats in the control group developed edema at the PI site at 1 h after decompression and continued to 72 h; however, only mild edema developed in the rats in the PC group, which gradually disappeared after 36 h. The average ulcer areas of the control and PC groups were (10.71 ± 13.15) mm^2^ and (2.76 ± 4.45) mm^2^ (*t* = 2.630, *p* = 0.015), respectively. The rats bathed with hot water developed fewer ulcers at 24, 36 and 72 h after decompression than those in the control group (Figure 2a). Although the incidences of ulcers had no statistical significance (Figure 2a), a considerable difference was observed in the areas of the ulcers at 24, 36, 48 and 72 h after decompression (all *p* < 0.05, Figure 2b); furthermore the severity of injury in the PC group was considerably relieved.

### 2.2. Skin Temperature and Moisture of PI Wounds

**Figure 3:**
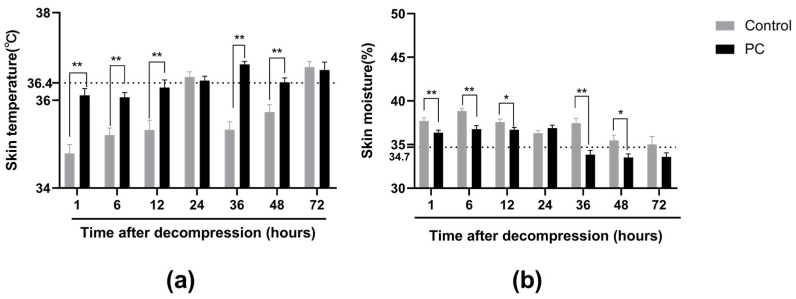
Skin temperature (**a**) and moisture (**b**) of PI wounds after decompression in two groups. Rat normal skin temperature was 36.4 ± 0.5 °C, and normal skin moisture was 34.7 ± 1.8%. * *p* < 0.05, ** *p* < 0.01. PC: preconditioning. The dotted line represents the normal value.

Before the experiment, we measured the dorsal skin temperature and skin moisture of the same rats without injuries. The normal rat skin temperature was 36.4 ± 0.5 °C, and the normal skin moisture was 34.7 ± 1.8%. At 1 and 72 h after decompression, the skin temperature of the control decreased and was lower compared with the PC group (*p* < 0.05: except 24 and 72 h, Figure 3a); however, it remained normal in the PC group. We further compared the skin moisture between the two groups. As shown in Figure 3b, the skin moisture of the two groups increased compared with the normal skin moisture in rats without injuries, and hydrotherapy resulted in skin moisture that was lower compared with the control group after decompression. Skin moisture became normal after 36 h in the PC group.

### 2.3. Hsp27 Expression of PI Wounds

**Figure 4:**
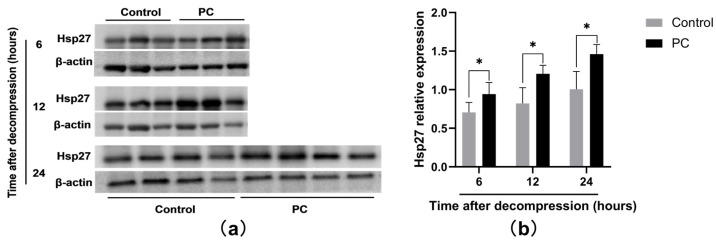
Hsp27 expression of PI wounds at 6, 12, and 24 h. (**a**) Western blot analysis of Hsp27 of the two groups. (**b**) Hsp27 relative expression of the two groups. * *p* < 0.05. PC: preconditioning.

To explore the possible molecular mechanisms responsible for the prevention of ulceration induced by hydrotherapy, we investigated the expression of Hsp27 of PI wounds at 6, 12, and 24 h after the compression was released. Figure 4a demonstrated Western blot analysis of Hsp27 of the two groups. In Figure 4b, the PC group showed a considerably higher expression of Hsp27 after decompression compared with the control group (*p* < 0.05).

## 3. Discussion

The development of PI in clinical nursing facilities is complicated and sometimes unavoidable. The treatment of ulcers is difficult, expensive, and has some adverse effects on the patient’s quality of life. Previous studies identified the efficacy of hydrotherapy in the prevention of enthesopathy and stress-related myocardial injury [30,31]. Moreover, heat shock preconditioning can effectively enhance cell survival and cell function in vitro in various culture environments [34]. In the present study, we investigated the possibility of using hydrotherapy preconditioning as a new method for PI prevention by using a hairless-rat PI model. Our results suggest that hydrotherapy preconditioning can effectively prevent the formation of ulcers in rats compared with the non-hydrotherapy group, reducing ulcer area and accelerating wound healing.

In our present study, considering a macroscopic perspective, we found that ulcers occurred at 24 h for the control, whereas they appeared after 36 h in the PC group, indicating that bathing with hot water can delay the occurrence of PI. Furthermore, the area of ulcer and its rate in the PC group was less than that in the control group at 24, 36, 48, and 72 h after decompression. These findings indicate that hydrotherapy preconditioning can effectively prevent ulcers. Heating caused the tissue to be stiffer, allowing less deformation [35]. In addition, increased temperature caused an increase in skin perfusion and an improvement in circulation, both locally and systemically. The possible mechanisms for these temperature-induced increases in blood flow are as follows: (1) temperature-mediated increases in capillary pressure, which increases perfusion; (2) temperature-induced-perfusion increases caused by changes in blood viscosity and erythrocyte membrane properties; and (3) the presence of a temperature-sensitive anterior capillary sphincter capable of changing flow resistance in response to (average) ambient temperatures. When combined with 100% humidity, the effects of increasing blood flow are more considerable. At a temperature of 42 °C and humidity of 100%, the largest increase in skin blood flow was observed [36,37]. When the humidity was higher, the skin heated up faster, and the blood circulation response became stronger. A moist heat method is more effective than dry heat for increasing skin blood flow. This process may contribute to the evaporation of sweat. For the rats bathed with hot tap water, their skin was not capable of losing heat by sweat because sweat does not evaporate. With the increase in blood flow in the skin, the possibility of developing ulcers decreased [38].

To explore hydrotherapy’s mechanisms of ulcer prevention, we evaluated the protein expression of Hsp27 by Western blot analysis. We found that the expression of Hsp27 in the PC group was considerably higher than that in the control group at 6, 12, and 24 h, indicating that hydrotherapy upregulated Hsp27.

An important aspect of the stress response is the potential to induce higher levels of stress tolerance and an increased resistance to subsequent damage from more than one type of stress [29]. Repeated mild-heat-stress-induced hormesis is beneficial in differentiation, wound healing, and angiogenesis [29]. During HSR, cells activate a signaling pathway that expresses Hsp. The hormetic response correlates with changes in the expression levels of Hsp27 [39]. Hsp27 is a member of a small HSP family and is constitutively expressed in cells. Its main functions include modulating the ability of cells to respond to several types of injury, such as heat shock and oxidative stress [40]. It can be rapidly activated at elevated temperatures. Mao et al. found that after heating with hot water, Hsp27 expression in tongue squamous cell carcinoma increased continuously for 12 h [41]. Similarly, the exposure of mesenchymal stem cells to high temperature (43 °C) was associated with an increased secretion of Hsp 27, which may have contributed to the increased cell survival [42]. The interaction between oxidative stress and apoptosis plays an important role in the formation of early PI [39]. After being subjected to oxidative stress, cells produce reactive oxygen species, various inflammatory cytokines, and other harmful substances, which leads to cell damage and PI. Our results show that hydrotherapy preconditioning relieves skin damage and reduces PIs. Geum et al. reported that heat shock preconditioning of neural progenitor cells at 43 °C for 3 h could reduce cell apoptosis [43]. Likewise, the short-term culture of cardiac progenitor cells at 42 °C reduces apoptosis, increases the functionality, and reduces the fibrosis of mouse ischemic myocardium [44]. These findings may contribute to an understanding of the upregulation of Hsps [43,44]. In our study, the expression of Hsp27 increased after hydrotherapy preconditioning. Hsp27 is involved in cellular protection in response to various stresses, including stress and heat shock [45]. It can reduce the production of harmful substances, such as reactive oxygen species. Furthermore, it can protect cells against an induction of cell death (including apoptosis and necrosis). Zou et al. reported that Hsp27 promotes vascular smooth muscle cell viability, suppresses cell apoptosis, and confers protection against oxidative stress [46], thus reducing the development of PIs.

There is increasing evidence that ischemia-reperfusion (I/R) injury is associated with the development of PIs [47]. I/R injury occurs after the ischemic tissue is relieved of pressure and blood perfusion is restored [48]. In the early stages of I/R injury, various events occur that could lead to PIs, such as endothelial cell injury, thrombus and edema formation, and the production of proinflammatory cytokines by infiltrating leukocytes and macrophages [49].

Previous studies have proved that Hsp27 has a significant protective effect on I/R injury in the liver [50], kidney [51], heart [52], and retina [53]. For example, an appropriately elevated temperature increases liver tolerance to I/R injury because of the increase in the expression of Hsp27 [50]. In our study, fewer occurrences of edema were observed in the PC group, which was consistent with a previous report that Hsp27 can relieve edema during acute pancreatitis [54]. We speculate that the relief of edema may be linked to the alleviation of I/R with increased Hsp27. Moreover, an inflammatory response after trauma exacerbates tissue edema and mediates secondary tissue damage [55]. The excessive release of inflammatory mediators and cytokines plays an important role in the occurrence of PIs [56]. Hsp27 participates in the immune response and reduces the infiltration of inflammatory cytokines [57]. Therefore, hydrotherapy preconditioning can effectively inhibit the inflammatory responses of the pressure tissue by increasing Hsp27 expression, regulating the cellular immune functions of the body, and reducing edema.

Before ulcers occurred, skin moisture levels in the two groups remarkably increased and and were considerably higher in the control compared with the PC group, which is consistent with our observations regarding edema. Skin moisture levels can be used as biophysical measurements to express skin defense [10,58]. Kim et al. found that the skin moisture values of early PIs were considerably higher than in those without injury [59]. Higher skin moisture levels are associated with an increased risk of developing ulcers [60,61]. Skin moisture values increase as the level of skin damage increases [61]. Moreover, the higher the skin moisture value, the higher the PI stage [10]. Tissue ischemia results in hypoxia and nutrient deprivation, and metabolites begin to deposit in tissues, thus increasing the permeability of damaged capillaries. This condition can be caused by the accumulation of interstitial fluid in the extracellular space caused by vascular leakage. Apoptosis, necrosis, and inflammation can also lead to elevated values [62]. Increased skin moisture levels contribute to maceration and skin breakdown [63], leading to skin damage by external forces [64]. Therefore, hydrotherapy preconditioning relieves edema by increasing the expression of Hsp27, leading to less skin moisture and reducing the ulcer area.

In addition, as shown in Figure 3a, skin temperature decreased in the control group at 1, 6, 36, and 48 h after decompression. The skin temperatures of the PC group had no considerable change compared with the rats’ normal temperatures; however, they were higher compared with the control group. Zhang et al. explored the relationship between I/R and thrombosis in skeletal muscle, and they found that the body demonstrates an inflammatory response after skeletal muscle I/R, which constitutes the three elements of thrombosis: slow blood flow, venous-wall damage, and hypercoagulability [65]. In the control group, thrombosis in capillaries eventually caused perfusion failure, which manifests as a decreased tissue temperature in rats. In rats that underwent hydrotherapy, less I/R injury happened with elevated Hsp27. Therefore, less thrombosis developed, and skin temperature remained basically unchanged. Generally, thrombosis is a considerable factor in pressure injury generation. Hence, hydrotherapy preconditioning can protect skin from being damaged by reducing thrombus and by increasing the expression of Hsp27.

**Figure 5:**
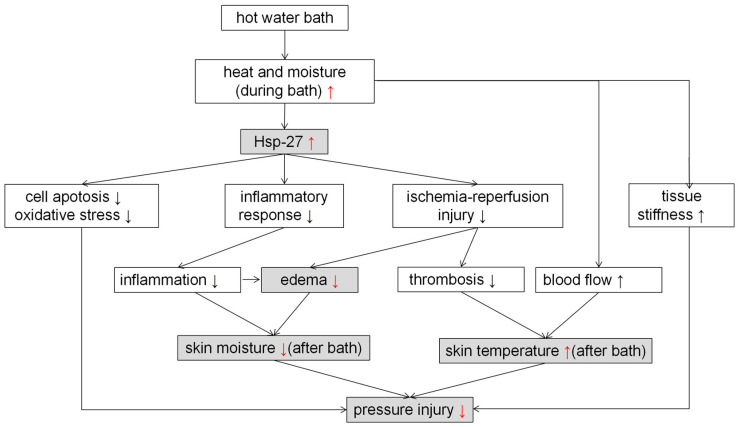
Effect of heat shock preconditioning on prevention of PI. The results of this study are marked as gray. References are in square parentheses. Red arrow (↑↓) means increase (↑) or decrease (↓) compared with the control group.

Consequently, as shown in Figure 5, though Hsp27 induction, hydrotherapy protection might prevent skin from being damaged, and the results are even more evident with increases in time. However, the mechanism of protection in Hsp27 regarding PIs has not been studied and thus requires further research.

## 4. Materials and Methods

### 4.1. Animals

Ten 7-week-old healthy male Wistar Yagi hairless rats with a weight of 250–290 g were obtained (HWY/Slc: SLC, Inc., Shizuoka, Japan). They were maintained on standard food and water. All the experiments were conducted in accordance with the recommendations in the *Guide for the Care and Use of Laboratory Animals of the National Institutes of Health*, and they were approved by the ethics committee of Animal Experiments of Nagano College of Nursing (No.2017-5).

### 4.2. Grouping

The rats were kept in an automatically controlled room (temperature, 21.4 ± 2.0 °C; humidity, 38.3% ± 7.6%) with a conventional light/dark cycle (L–D: 12–12 h). All the rats were fed with a standard laboratory diet with free access to water for 1 week. The rats were randomly divided into two groups by using the random number table generated by Excel software (Microsoft Office 2019, Microsoft Corporation). The treatment group was named preconditioning (PC). The other group without preconditioning was the control. Five rats were included in each group.

### 4.3. Protocol of The Study Design

**Figure 6:**
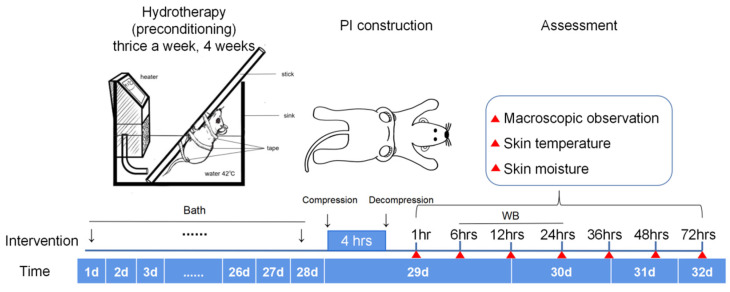
Protocol of the study design. PI: pressure injury. WB: Western blot.

Figure 6 shows the protocol of the study design. The PC group underwent hydrotherapy thrice a week at 08:00 a.m. for 4 weeks. The day after the last bath, PI models were constructed at 08:00 a.m. After decompression for 1, 6, 12, 24, 36, 48, and 72 h, skin appearance, temperature, and moisture of PI sites were observed. The measurement sites were consistently managed by marking them with an insoluble marking pen for a high uniformity in the measurement. Skin samples of PI wounds were obtained at 6, 12, and 24 h after decompression for Hsp27 analysis.

### 4.4. Preconditioning Group

Before being placed into water, the hairless rats were anesthetized with an intraperitoneal injection of a mixture of medetomidine hydrochloride (0.15 mg/kg Domitor, Zenoaq, Fukushima, Japan), midazolam hydrochloride (2.0 mg/kg; Dormicaum; Astellas Pharma, Tokyo, Japan), and butorphanol (2.5 mg/kg; Vetorphale; Meiji Seika Pharma Co., Ltd., Tokyo, Japan). Thereafter, the rats were tied onto a wooden stick with three cloth tapes (Battlewin, NICHIBAN, Tokyo, Japan, Figure 6). The rats were placed into a hot tap water-filled tank (EYELA NTT 2200, 30 cm × 45 cm × 15 cm) and the water was heated by a heater to maintain the water temperature at 42 °C for 15 min. During bathing, the respiratory status was observed. The rats underwent hydrotherapy thrice a week for 4 weeks. After 4 weeks of bathing, the PI models were constructed, and the PI wounds were observed at 1, 6, 12, 24, 36, and 72 h after pressure was released.

### 4.5. Control Group

As a control group, the rats were anesthetized three times a week without a bath by using the same process as for the PC group. The PI wounds were observed by utilizing the same method used for the PC group.

### 4.6. Construction of PI Rat Models

The PI rat model was adopted using magnetic compression focusing on I/R injury for the formation of skin ulcerations [66,67]. PI rat models were developed as described in a previous study [68]. The dorsal skin was gently pulled up and placed between 2 round magnetic disks measuring 10 mm and 3 mm in diameter and thickness respectively, with an average of 3000 Gauss magnetic force (NE05, Niroku Seisakusho Co., Ltd., Kobe, Japan). This process created a compressive pressure of 135 mmHg between the two magnets. The pressure between the two magnetic disks can lead to pressure on the skin and PI models were constructed because of local ischemia. We applied pressure for 4 h and created 4 PI sites in each rat.

### 4.7. Macroscopic Observation of PI

Photos of PI wounds were obtained using an AM313 digital camera (Optio WG-3 PENTAX, Japan) before bathing. The distance between the camera and the PI wounds was fixed at 20 cm, and the focal distance was four times the previous value. The photos were uploaded on NIH Image J ver.1.47 (National Institutes of Health, Bethesda, MD http://imagej.nih.gov/ij/download.html (accessed on 20 July 2020), and the average area of the PI wounds was measured.

### 4.8. Assessment of Skin Temperature

Thermography is used to measure the spectrum of the infrared energy emitted by the skin and depict it in an image, where varied colors are equivalent to skin temperature changes. In our study, FLIR i3 infrared-ray thermography non-contact thermometer (FLIR i3 Thermal Imager, FLIR Systems, Inc. Wilsonville, OR, USA) was used to detect the surface temperatures in the PI sites before bath. The distance between the camera and the PI sites was fixed at 48 cm. After the photos were obtained, images were analyzed using the FLIR Tools software. Temperature measurements were conducted in an air-conditioned environment (average room temperature: 22 °C). For the analysis of the tissue temperature, the thermometer was applied over the PI sites, and the measurement was conducted thrice. The mean value was accepted.

### 4.9. Skin Moisture Measures

The DM-R2 skin moisture scanner (Panasonic Co., Tokyo, Japan) was used to detect skin moisture levels. The moisture levels was in the units of 1 and 100%. A small wand was placed on the skin surface for 5 s to read the value, and the higher value, the higher the skin moisture level. The measurement was performed thrice, and the mean values were recorded.

### 4.10. Protein Preparation and Western Blot Analysis

The protein expression of Hsp27 was examined. In order to examine Hsp27 expression, we conducted the same experiment using another 6 hairless rats according to the design protocol (3 rats in each group). We obtained skin samples from the PI wounds by surgery biopsy from different rats at 6, 12, and 24 h after decompression for protein analysis. The samples were snap frozen immediately following resection. The tissue was homogenized in ice-cold RIPA, and then centrifuged at 12,000× *g* for 10 min at 4 °C. The supernatant containing the total cell protein was extracted for protein concentration assay. Samples were separated on 10% sodium dodecyl sulfate-polyacrylamide gels, and then transferred onto a nitrocellulose membrane. Each well was loaded with approximately 30 μg of soluble protein. Placental tissue, which expresses Hsp27, was used as a reference sample and positive control on each gel. The primary antibody (HSP 27, Santa Cruz) was used as 1:1000. The second antibody was horseradish peroxidase-conjugated donkey anti-mouse secondary antibody (Jackson, 1:5000). Proteins were visualized using the ECL detection system (Thermo Fisher Scientific). Bands were scanned using a GE imaging densitometer. Band densities were expressed relative to the density of the internal placental control sample included on every well by using the ImageJ software.

### 4.11. Statistical Analysis

IBM SPSS Statistics version 22.0 software (IBM, Armonk, NY, USA) was used for statistical analysis, and GraphPad Prism 5 (GraphPad Software, La Jolla, CA, USA) was used for data visualization. All values are expressed as mean ± standard deviation, and Student’s *t* *t*-test was conducted to test changes in the PI sites over time between the two groups. Two-tailed *p*-value less than 0.05 was considered statistically significant.

## 5. Conclusions

Heat shock preconditioning considerably attenuated PI events and reduced the ulcer area in rats, which we confirmed by recording decreased ulcer rates and areas. Hsp27 was involved in hydrotherapy’s protective effects against PI in rats. Our study is of interest to the PI-care clinician because it provides evidence for the benefits of hydrotherapy preconditioning in PI prevention. This method is simple to implement and is a cost-effective preventive measure.

## 6. Limitations

This study has some limitations. First, we applied hydrotherapy to rats, which showed high efficacy in PI prevention. However, whether the frequency and temperature are suitable for humans remains unclear. Moreover, although the effects of Hsp27 on PI prevention has been discussed, future histopathology experiments are still needed to verify the relationships between thrombosis, inflammation, and other lesions. Furthermore, in addition to Hsp27, other heat shock proteins or factors may participate in PI prevention during hydrotherapy. Finally, the tap water we used may contain various minerals that might affect the protection of skin from being damaged. Therefore, additional studies are needed to explore further factors.

## Data Availability

The data presented in this study are available on request from the corresponding author.
